# Anatomical classification of advanced biliary tract cancer predicts programmed cell death protein 1 blockade efficacy

**DOI:** 10.3389/fphar.2024.1375769

**Published:** 2024-08-30

**Authors:** Lingli Huang, Fang Wang, Fenghua Wang, Qi Jiang, Jinsheng Huang, Xujia Li, Guifang Guo

**Affiliations:** ^1^ VIP Department, Sun Yat-Sen University Cancer Center, Guangzhou, China; ^2^ State Key Laboratory of Oncology in South China, Sun Yat-Sen University Cancer Center, Guangzhou, China; ^3^ Collaborative Innovation Center for Cancer Medicine, Sun Yat-Sen University Cancer Center, Guangzhou, China; ^4^ Guangdong Provincial Clinical Research Center for Cancer, Sun Yat-Sen University Cancer Center, Guangzhou, China; ^5^ Department of Oncology, The First Affiliated Hospital of Sun Yat-Sen University, Guangzhou, China; ^6^ Department of Medical Oncology, Sun Yat-sen University Cancer Center, Guangzhou, China

**Keywords:** biliary tract cancer, immunotherapy, programmed cell death protein 1, intrahepatic cholangiocarcinoma, extrahepatic cholangiocarcinoma

## Abstract

**Background:**

Immune checkpoint blockade (ICB)-based immunotherapy has inspired new hope for advanced biliary tract cancer (BTC) treatment; however, there are no prior studies that primarily focus on different anatomical types of unresectable BTCs reacting differently to ICB.

**Methods:**

We retrospectively collected data on advanced BTC patients who received anti-programmed cell death protein 1 (anti-PD1) therapy from two affiliated hospitals of Sun Yat-Sen university. The effects of anti-PD1 were compared for different anatomical sites. The GSE32225 and GSE132305 datasets were used to further analyze differences in the immune microenvironments between intrahepatic cholangiocarcinoma (ICC) and extrahepatic cholangiocarcinoma (ECC).

**Results:**

A total of 198 advanced BTC patients were enrolled in this study, comprising 142 patients with ICC and 56 with other cancer types (“Others” group), including ECC and gallbladder cancer. In the anti-PD1 treated patients, the ICC group (n = 90) achieved longer median progression-free survival (mPFS) (9.5 vs. 6.2 months, *p* = 0.02) and median overall survival (mOS) (15.1 vs. 10.7 months, *p* = 0.02) than the Others group (n = 26). However, chemotherapy did not show different effects between the two groups (mOS: 10.6 vs. 12.1 months, *p* = 0.20; mPFS: 4.9 vs. 5.7 months, *p* = 0.83). For the first-line anti-PD1 therapy, the ICC group (n = 70) achieved higher mOS (16.0 vs. 11.8 months, *p* = 0.04) than the Others group (n = 19). Moreover, most chemokines, chemokine receptors, major histocompatibility complex molecules, immunostimulators, and immunoinhibitors were stronger in ICC than ECC; furthermore, CD8^+^ T cells and M1 macrophages were higher in ICC than ECC for most algorithms. The immune differential genes were mainly enriched in antigen processing and presentation as well as the cytokine receptors.

**Conclusions:**

This study shows that the efficacy of anti-PD1 therapy was higher in ICC than in other types of BTCs. Differences in the immune-related molecules and cells between ICC and ECC indicate that ICC could benefit more from immunotherapy.

## Introduction

Biliary tract cancers (BTCs) can be broadly classified under intrahepatic cholangiocarcinoma (ICC), extrahepatic cholangiocarcinoma (ECC), and gallbladder cancer (GBC) depending on the anatomical primary sites (Scott et al., 2022). BTC has low incidence but causes highly lethal malignancies (Valle et al., 2021). Very few patients with BTC have the chance to undergo radical surgical therapy and there is a high relapse rate after surgery (Siegel et al., 2021), with the 5-year survival rates ranging from 20% to 35% (Wang et al., 2013). Most patients with BTC are diagnosed at advanced stages, and patients with surgically unresectable cancer have a median survival of approximately 12 months (Valle et al., 2010; Valle et al., 2014).

Cisplatin with gemcitabine has been used as the standard of treatment for patients with unresectable BTCs (Valle et al., 2010). Unfortunately, for people with metastatic BTCs who suffer from progression even with chemotherapy, there is no consensus on chemotherapy-resistant BTC (Lin et al., 2020a). In addition, even patients who initially respond well to treatment eventually show cancer progression, for which subsequent effective treatments are lacking. Therefore, the need for additional treatment options continues to exist.

Immune checkpoint inhibitors (ICIs), including programmed cell death protein 1 (PD1) and PD ligand 1 (PDL1), have emerged as promising options for treating various solid tumors (Hamanishi et al., 2016). Increasing evidence indicates that cancer immunotherapy is a promising treatment against recurrence and metastasis (Wang et al., 2019). Many phase 2 drug studies have demonstrated that immunotherapy, including anti-PD1, anti-PDL1, and anti-CTLA4, combined with chemotherapy shows promising efficacy and acceptable safety in advanced BTC patients (Vogel et al., 2022; Kim et al., 2020; Yarchoan et al., 2021a; Chen et al., 2020).

Results from the KEYNOTE-158 study showed that the median overall survival (mOS) and median progression-free survival (mPFS) for patients receiving pembrolizumab were 7.4 and 2.0 months, respectively, with the objective response rate (ORR) being only 6.6% (Piha-Paul et al., 2020; Marabelle et al., 2020). Kim et al. showed that the mOS and mPFS of nivolumab monotherapy in the treatment of advanced BTCs were 14.24 and 3.68 months, respectively, and that the mPFS was shorter for traditional chemotherapy (3.68 vs. 8.0 months) (Kim et al., 2020). IMMUCHEC is an ongoing multicenter phase 2 study to assess the efficacies of dual immunotherapy with and without chemotherapy in advanced BTCs (Vogel et al., 2022). The mOS for the dual immunotherapy and chemotherapy combination group was 22.7 months. However, the rate of adverse effects above grade 3 was 86%. Moreover, the overall survival (OS) and progression-free survival (PFS) for PDL1 or CTLA-4 combined with chemotherapy were worse than those for standalone chemotherapy (Vogel et al., 2022). Currently, TOPAZ-1 and KEYNOTE-966 are two multicenter clinical trials aimed at assessing the efficacies of anti-PDL1/anti-PD1 combined with chemotherapy as first-line treatments for advanced BTC that have shown promising results (Oh et al., 2022a; Kelley et al., 2023). Yarchoan et al. (2021a) showed that anti-PDL1 combined with MEK inhibitors in BTC offers more efficacy and advantages to ICC patients, with the mPFS increasing from 1.87 to 3.65 months compared to monotherapy with atezolizumab. Interestingly, in a study that enrolled only advanced ICC patients, the ORR and disease control rate (DCR) for anti-PD1 combined with chemotherapy and targeted therapy were 80% and 93.3%, respectively; similarly, the mPFS rate was 10.0 months, and the 12-month OS rate was 73.3% (Jian et al., 2021), which were significantly better than those observed for conventional chemotherapy and other immunotherapy studies, indicating that ICC may benefit more from immunotherapy. Some reports have also shown that different sites of BTCs in patients have markedly heterogeneous clinical outcomes because of their molecular characteristics (Yoon et al., 2021; Chen et al., 2021). Given the current progress with immunotherapy in advanced BTCs and inconsistent results from different studies (Yarchoan et al., 2021a; Oh et al., 2022a; Yoon et al., 2021; Chen et al., 2021; Wang et al., 2022; Oh et al., 2022b), it is of interest to explore whether different anatomical types of unresectable BTCs would respond differently to immune checkpoint blockade (ICB).

In our present work, we contrast the effects of anti-PD1 therapy between different anatomical classifications of advanced BTCs in Chinese multicenter cohorts. Furthermore, we explore the intrinsic mechanisms of the processes using information obtained from public databases.

## Materials and methods

### Patients

We retrospectively screened patients from the Sun Yat-Sen University Cancer Center and the First Affiliated Hospital of Sun Yat-Sen University in China; these patients were diagnosed with advanced BTC between January 2014 and September 2020. The following inclusion criteria were used during screening: histopathologic diagnosis of ICC, ECC, or GBC; unresectable locally advanced or metastatic BTC; chemotherapy alone or combined with anti-PD1 regimen for more than two cycles; complete radiographic evaluation. The exclusion criteria were as follows: other primary malignant tumors in addition to BTC; radiotherapy before anti-PD1 treatment; uncontrolled intercurrent illness; surgical resection of metastatic lesions. A total of 198 patients were included after screening, and the study protocols were approved by the ethics committees of Sun Yat-Sen University Cancer Center (B2020-190-01) and the First Affiliated Hospital of Sun Yat-Sen University ([2022]069), with the exemption of informed consent. The study was conducted retrospectively without affecting the interests of the patients and adhered to the guidelines of the Declaration of Helsinki.

### Data extraction and response evaluation

An IRB-approved protocol was followed to search the electronic medical records of the patients for the treatment options, clinical characteristics, and efficacy evaluations. Two oncologists examined the treatment efficacies in accordance with the Response Evaluation Criteria in Solid Tumors (RESIST) criteria (1.1) (Armato and Nowak, 2018). Any inconsistencies in the results were solved by consensus. The effects were evaluated by measuring the changes in the target lesions through imaging. The ORR, DCR, PFS, and OS values were used to assess the treatment efficacy. PFS refers to the time between receiving immunotherapy or chemotherapy and the earliest response between progressive disease or mortality; OS is defined as the time range from registration into the study to the date of death. A standard follow-up was conducted for all participants, and the total follow-up period was more than 24 months unless death occurred. The last follow-up visit for the participants was October 2022 or death.

### Treatments

The beginning and end dates of treatment, initial dose, and dose modifications were systematically collected. The anti-PD1 drugs included pembrolizumab, sintilimab, toripalimab, camrelizumab, and tirelizumab. The immunotherapy treatment regimen included administration of anti-PD1 in combination with an antiangiogenic or chemotherapy or transarterial chemoembolization or hepatic arterial infusion chemotherapy.

### Data acquisition and processing

The ICC (GSE32225) and ECC (GSE132305) datasets were obtained from the Gene Expression Omnibus (GEO) database before being log normalized (log2x+1) and merged. The “limma” R package was used for differential expression analysis, where the log2Foldchange parameter was set to 0.5 and adjusted *p*-value was 0.05. The intersection of the differentially expressed genes and immune-related genes in the ImmPort dataset (https://www.immport.org/shared/genelists) was selected, and a volcano map was drawn to show the differentially expressed immune-related genes.

### Immune predictions

The stromal, immune, and ESTIMATE scores of the ICC and ECC samples in the two datasets were calculated using the ESTIMATE algorithm in the “estimate” R package (Yoshihara et al., 2013). The tumor immune dysfunction and exclusion (TIDE) (http://tide.dfci.harvard.edu/) algorithm was used to predict the potential ICB therapy responses (Jiang et al., 2018). TISIDB (http://cis.hku.hk/TISIDB) was used to compare the differences in the immune-related molecules, including immunostimulators, immunoinhibitors, major histocompatibility complex (MHC) molecules, chemokines, and chemokine receptors (Ru et al., 2019).

### Tumor-infiltrating immune cells

We used five algorithms to calculate the differences in the tumor-infiltrating immune cells between the GSE32225 and GSE132305 datasets. Xcell (http://xcell.ucsf.edu/) (Aran et al., 2017) and MCP-counter (http://github.com/ebecht/MCPcounter/) (Becht et al., 2016) were used as the quantification methods for the marker-gene-based tumor-infiltrating immune cells, while CIBERSORT (https://cibersort.stanford.edu/) (Newman et al., 2015), TIMER (https://cistrome.shinyapps.io/timer/) (Li et al., 2017), and quanTIseq (http://icbi.i-med.ac.at/software/quantiseq/doc/index.html) (Finotello et al., 2019) were used as the quantification methods for the tumor-infiltrating immune cells based on expression feature deconvolution of the cell mixture.

### Statistical analysis

The cutoff date (October 2022) for our report was used to generate the summary of baseline characteristics and efficacy assessments. The Cox proportional hazards model and Kaplan–Meier method were used to estimate the PFS, OS, 6-month and 12-month OS (PFS), and hazard ratios (HRs); the log-rank test was then used for comparative analyses. The Chi-square and Fisher’s exact tests were used to compare the categorical variables, while the Student’s t-test was used for the continuous variables. A value of *p* < 0.05 was set as the statistically significant threshold. SPSS software (SPSS Statistics 25) and R4.2.2 were used for the statistical analyses.

## Results

### Clinical characteristics of advanced BTC patients

A total of 198 patients with advanced BTCs were enrolled in this study and consisted of 142 cases with ICC as well as 56 cases of ECC and GBC (referred to as “Others”). The basic clinical characteristics of the participants are shown in Table 1. At the time of diagnosis, 33.8% (48/142) of the ICC patients had a history of hepatitis B virus (HBV) infection, while this value was 14.3% (8/56) for the Others group. About 61.3% of the ICC patients had distant or intrahepatic metastases while receiving chemotherapy or immunotherapy, and 55.4% of the patients in the Others group had metastases. Moreover, 41.4% (82/198) of the patients received standalone chemotherapy while the remaining 58.6% (116/198) of patients received chemotherapy along with anti-PD1 therapy. The characteristics of the BTC patients receiving anti-PD1 therapy are shown in Table 2. There were no statistically significant differences in the age, gender, pathological grade, clinical stage, metastasis, or HBV infection between patients in the ICC and Others groups. The baseline characteristics of the ICC, ECC, and GBC patients receiving anti-PD1 combination therapy are documented in Supplementary Table S4.

**TABLE 1:** Clinical characteristics of advanced biliary tract cancer (BTC) patients.

	ICC (n = 142)	Others (n = 56)	*p*-value
Gender (%)			0.577
Female	53 (37.3)	24 (42.9)	
Male	89 (62.7)	32 (57.1)	
Age in years (95% CI)	55.00 (48.00–63.00)	58.00 (51.75–66.00)	0.061
HBV infection (n, %)			0.023
Positive	48 (33.8)	8 (14.3)	
Negative	90 (63.4)	46 (82.1)	
Unknown	4 (2.8)	2 (3.6)	
Pathological grade (n, %)			0.365
Low	31 (21.8)	12 (21.4)	
Low to moderate	32 (22.5)	18 (32.1)	
Moderate	39 (27.5)	17 (30.4)	
Advanced	2 (1.4)	1 (1.8)	
Unknown	38 (26.8)	8 (14.3)	
Clinical stage (n, %)			0.708
IIIB or IIIC	45 (31.7)	20 (35.7)	
IV	97 (68.3)	36 (64.3)	
Metastatic (n, %)			0.547
Yes	87 (61.3)	31 (55.4)	
No	55 (38.7)	25 (44.6)	
Prior surgery (n, %)			0.185
Yes	62 (43.7)	31 (55.4)	
No	80 (56.3)	25 (44.6)	
Treatment (n, %)			0.083
Chemotherapy alone	52 (36.6)	30 (53.6)	
Anti-PD1 as first-line therapy	70 (49.3)	19 (33.9)	
Anti-PD1 as second-line therapy	20 (14.1)	7 (12.5)	

ICC, intrahepatic cholangiocarcinoma.

Others (including extrahepatic cholangiocarcinoma and gallbladder cancer).

**TABLE 2:** Clinical characteristics of advanced biliary tract cancer (BTC) treated with anti-PD1.

	ICC (n = 90)	Others (n = 26)	*p*-value
Gender (%)			1.000
Female	34 (37.8)	10 (38.5)	
Male	56 (62.2)	16 (61.5)	
Age in years (95% CI)	55.00 (47.25–62.75)	59.50 (54.00–66.00)	0.059
HBV infection (n, %)			0.161
Positive	32 (36.4)	5 (19.2)	
Negative	56 (63.6)	21 (80.8)	
Pathological grade (n, %)			0.39
Low	16 (25.0)	2 (9.5)	
Low to moderate	18 (28.1)	8 (38.1)	
Moderate	29 (45.3)	10 (47.6)	
Advanced	1 (1.6)	1 (4.8)	
Unknown	38 (26.8)	8 (14.3)	
Clinical stage (n, %)			0.561
IIIB or IIIC	37 (41.1)	13 (50.0)	
IV	53 (58.9)	13 (50.4)	
Metastatic (n, %)			0.504
Yes	47 (52.2)	11 (42.3)	
No	43 (47.8)	15 (57.7)	
Anti-PD1 treatment			0.607
First line	70 (77.8)	19 (73.1)	
Second line	20 (22.2)	7 (26.9)	

ICC, intrahepatic cholangiocarcinoma.

Others (including extrahepatic cholangiocarcinoma and gallbladder cancer).

### Efficacy assessments of anti-PD1 therapy in different types of advanced BTCs

**FIGURE 1:**
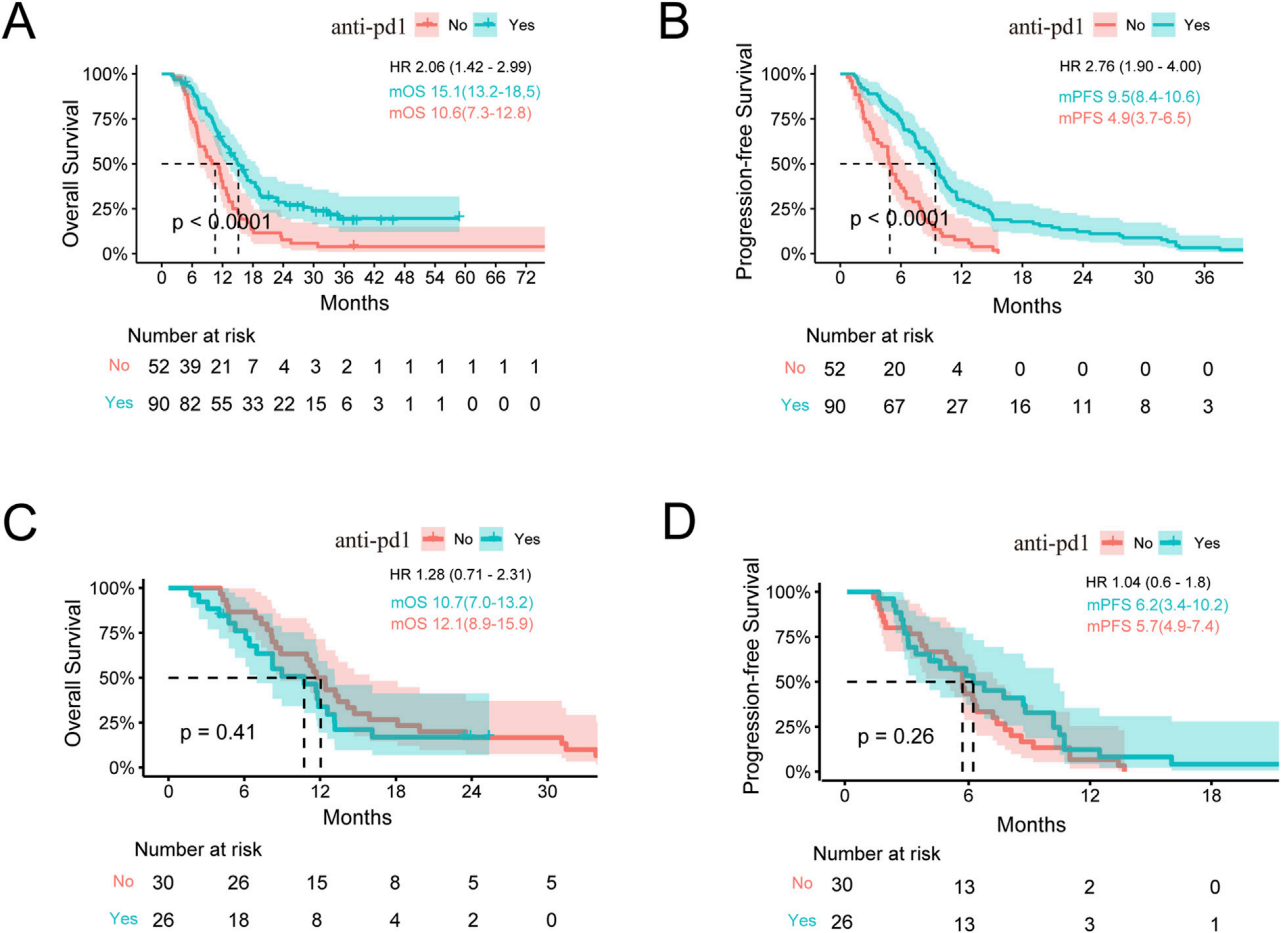
Survival analysis stratified by immunotherapy. **(A,B)** Kaplan–Meier estimates of the OS and PFS for anti-PD1 vs. no anti-PD1 therapy in ICC. **(C,D)** Kaplan–Meier estimates of the OS and PFS for anti-PD1 vs. no anti-PD1 in the Others group. OS, overall survival; PFS, progression-free survival; ICC, intrahepatic cholangiocarcinoma; Others, extrahepatic cholangiocarcinoma and gallbladder cancer; mOS, median overall survival; mPFS, median progression-free survival; HR, hazard ratio. Note: HR is the ratio of ECC to ICC.

**FIGURE 2:**
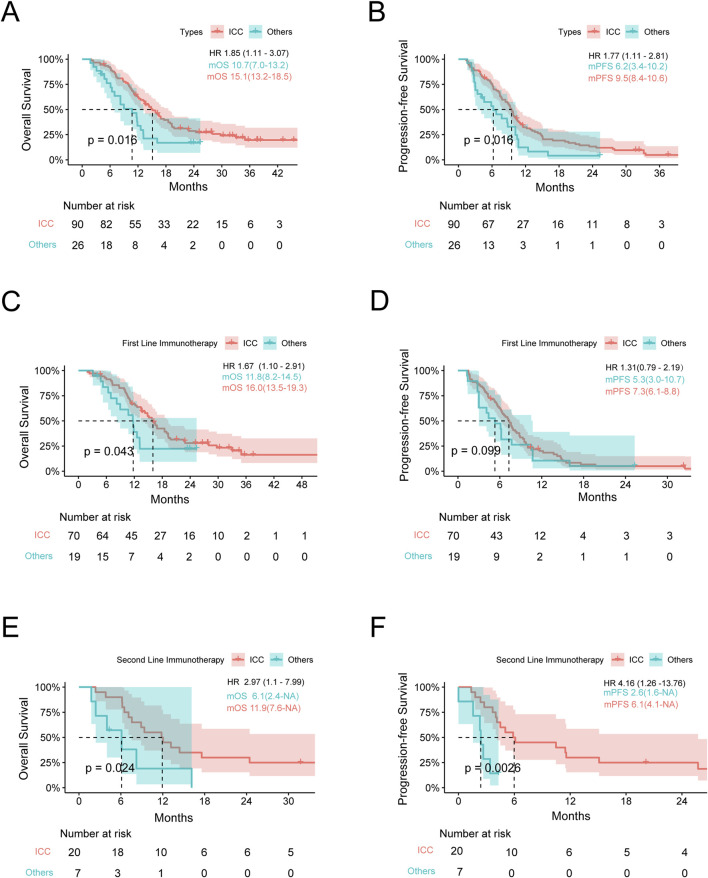
Survival analysis stratified by the tumor location. **(A,B)** Kaplan–Meier estimates of the OS and PFS for anti-PD1 therapy in the ICC and Others groups. **(C,D)** Kaplan–Meier estimates of the OS and PFS for first-line anti-PD1 therapy in the ICC and Others groups. **(E,F)** Kaplan–Meier estimates of the OS and PFS for second-line anti-PD1 therapy in the ICC and Others groups. OS, overall survival; PFS, progression-free survival; ICC, intrahepatic cholangiocarcinoma; Others, extrahepatic cholangiocarcinoma and gallbladder cancer; mOS, median overall survival; mPFS, median progression-free survival; HR, hazard ratio. Note: HR is the ratio of ECC to ICC.

We compared the efficacies of chemotherapy combined with anti-PD1 treatment vs. chemotherapy alone in advanced BTCs and showed that the latter was more effective (mOS: 13.2 vs. 11.2 months, *p* < 0.001; mPFS: 6.2 vs. 5.1 months, *p* < 0.001) (Supplementary Figures S1A, B). To compare the responses to immunotherapy from different anatomical sites of the subjects with or without another therapy, we divided the participants into two groups as ICC and Others. In patients with ICC, the OS and PFS when receiving immunotherapy were significantly better than those receiving non-immunotherapy treatments (mOS: 15.1 vs. 10.6 months, *p* < 0.001; mPFS: 9.5 vs. 4.9 months, *p* < 0.001), and HRs for OS and PFS were 2.06 (95% confidence interval (CI): 1.42–2.99) and 2.76 (95% CI: 1.90–4.00), respectively (Figures 1A, B). In the Others group, the patients receiving immunotherapy fared as well as those receiving chemotherapy alone (mOS: 10.7 vs. 12.1 months; mPFS: 6.2 vs. 5.7 months, *p* > 0.05 for both OS and PFS) (Figures 1C, D). To this end, we further studied the differences in anti-PD1 effects between the ICC and Others groups. Interestingly, among all the patients receiving anti-PD1, the ICC group achieved a mOS of 15.1 months, while the Others group achieved a mOS of 10.7 months (HR: 1.85, 95% CI: 1.11–3.07, *p* = 0.02); the ICC group achieved a mPFS of 9.5 months, while the Others group achieved a mPFS of 6.2 months (HR: 1.77, 95% CI: 1.11–2.81, *p* = 0.02) (Figures 2A, B; Supplementary Table S1). In the cohort receiving chemotherapy alone, the clinical outcomes of the two groups were compared; there were no significant differences in the mOS and mPFS values between the ICC and Others groups (mOS: 10.6 vs. 12.1 months, *p* = 0.20; mPFS: 4.9 vs. 5.7 months, *p* = 0.83) (Supplementary Figure S1C, D). In patients receiving anti-PD1 treatment, the ORR was 31.1% (95% CI: 21.8%–41.7%) and DCR was 85.6% (95% CI: 76.6%–92.1%) for the ICC group, with 2 complete response (CR), 26 partial response (PR), 49 stable disease (SD), and 13 progressive disease (PD) instances. Meanwhile, for the Others group, the ORR was 19.2% (95% CI: 6.6%–39.4%) and DCR was 80.8% (95% CI: 60.6%–93.4%), with 5 PR, 16 SD, and 5 PD cases. There were no significant differences in the ORR and DCR between the two groups (*p* = 0.35 and *p* = 0.77, respectively) (Supplementary Table S1).

### Efficacy evaluations with different lines of anti-PD1 therapy for advanced BTCs

To compare the efficacies with different lines of anti-PD1 therapy between the ICC and Others groups, we divided the anti-PD1 therapy cohort into first and second lines. Based on survival analysis of the first-line anti-PD1 therapy, the OS of the ICC group was better than that of the Others group, with mOS of 16.0 months (95% CI: 13.5–19.3) vs. 11.8 months (95% CI: 8.2–14.5) and *p* = 0.04; the mPFS was longer in the ICC cohort, while no statistically significant difference was found between the two cohorts at 7.3 months (95% CI: 6.1–8.8) vs. 5.3 months (95% CI: 3.0–10.7) and *p* = 0.10. The 6-, 12-, and 18-month survival rates of the ICC group were 60.0%, 15.7%, and 5.7%, while those of the Others group were 42.1%, 10.5%, and 5.3%, respectively. With regard to tumor shrinkage evaluation, the ORR was 30.0% (95% CI: 19.6%–42.1%) and DCR was 85.7% (95% CI: 75.3%–92.9%) for the ICC group, with 1 CR, 20 PR, 39 SD, and 10 PD cases; similarly, for the Others group, the ORR was 15.8% (95% CI: 3.4%–39.6%) and DCR was 78.9% (95% CI: 54.4%–93.9%), with 3 PR, 12 SD, and 4 PD cases (ORR: *p* = 0.26, DCR: *p* = 0.49) (Figures 2C, D; Supplementary Table S2).

Based on survival analysis of the second-line anti-PD1 therapy, the mOS values of the ICC and Others groups were 11.9 and 6.1 months, respectively (*p* = 0.02). Similarly, the mPFS values of the two groups were 6.1 and 2.6 months (*p* < 0.01). With regard to tumor shrinkage evaluation, the ORR was 30.0% (95% CI: 11.9%–54.3%) and DCR was 85.0% (95% CI: 63.1%–96.8%) for the ICC group, with 1 CR, 5 PR, 11 SD, and 3 PD cases; similarly, for the Others group, the ORR was 28.5% (95% CI: 3.7%–71.0%) and DCR was 85.7% (95% CI: 42.1%–99.6%), with 2 PR, 4 SD, and 1 PD cases (ORR: *p* = 1.00, DCR: *p* = 1.00) (Figures 2E, F; Supplementary Table S3).

We further analyzed the efficacies of anti-PD1 therapy in patients with ICC, ECC, and GBC. The results indicated that the OS of ICC patients was superior to those of the ECC and GBC patients (*p* = 0.049). However, the PFS was not statistically significant (*p* = 0.066), likely owing to the small sample size (Supplementary Figure S2).

### Prediction of ICB responses based on anatomical classification

**FIGURE 3:**
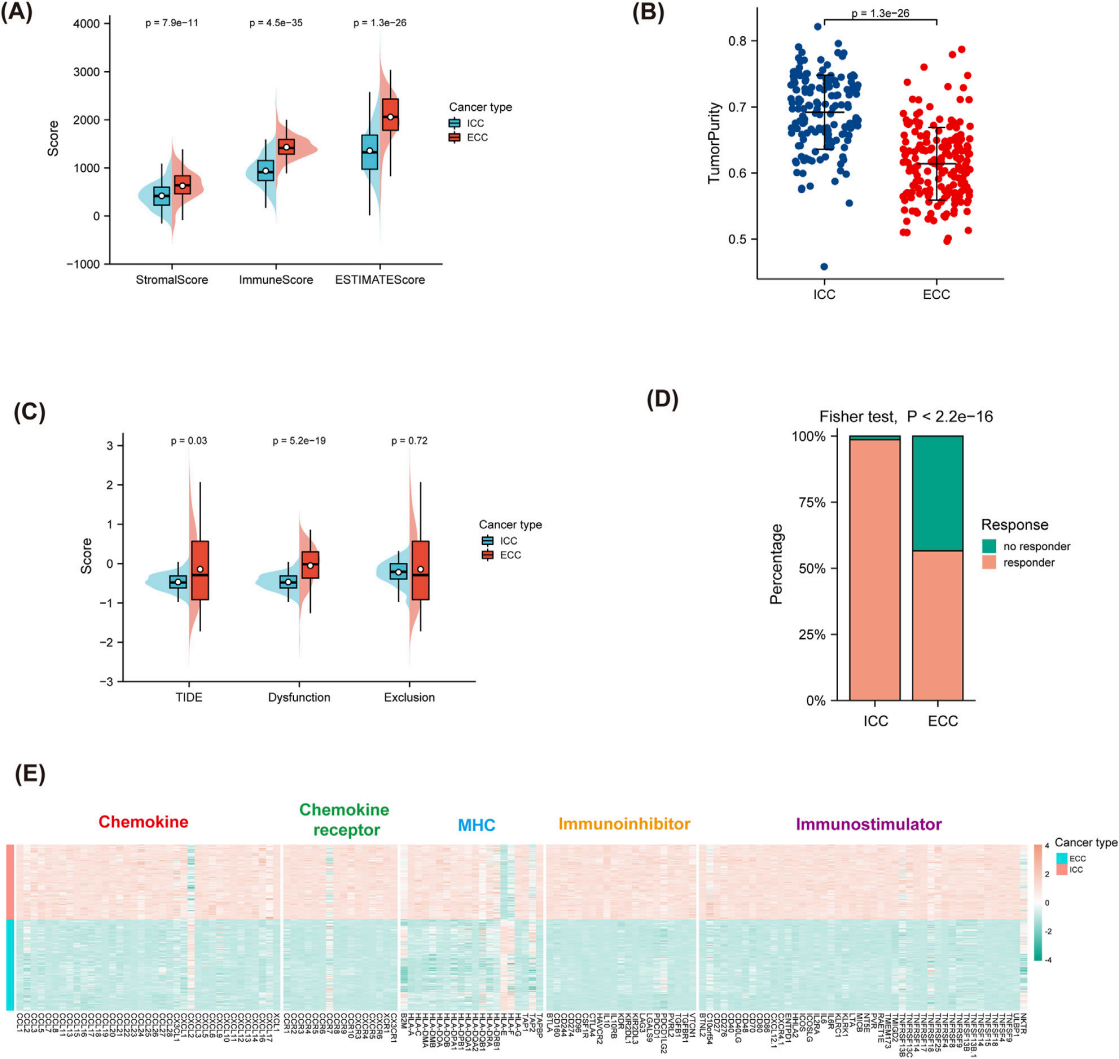
Prediction of ICB responses based on anatomical classification. **(A,B)** ESTIMATE scores for predicting the responses for ICB and tumor purity. **(C,D)** TIDE scores for predicting the responses for ICB and the response percentages. **(E)** Differences in immune-related molecules between ICC and ECC, including immunostimulators, immunoinhibitors, MHC molecules, chemokines, and chemokine receptors. ICC, intrahepatic cholangiocarcinoma; ECC, extrahepatic cholangiocarcinoma; MHC, major histocompatibility complex.

By analyzing clinical cases of BTCs, we found that patients diagnosed with ICC had better responses to immunotherapy than those diagnosed with other types of BTCs. To further understand the reasons for the high response rate of ICC to ICB therapy, two datasets from the GEO database, namely GSE32225 (for ICC) and GSE132305 (for ECC), were selected for further analyses. The matrices of the two datasets were extracted. ESTIMATE and TIDE were used to predict the responses of both groups to ICB. The results in Figures 3A, B show that the stromal, immune, and ESTIMATE scores for ICC were all lower than those for ECC; furthermore, the tumor purity was higher in ICC than ECC. Conversely, the results in Figures 3C, D show that the TIDE and dysfunction scores were lower in ICC than ECC, while the exclusion scores had no statistically significant difference. Considering that the TIDE prediction scores account for both T-cell dysfunction and T-cell rejection whereas most other biomarkers address only one of these factors, we believe that the TIDE prediction score might be superior to the other scores. Therefore, ICC was considered to have better immune effects than ECC, which is consistent with the results observed in clinical patients.

### Differences in immune-related molecules between ICC and ECC

TISIDB was used to calculate the differences in the immune-related molecules between ICC and ECC. The results shown in Figure 3E indicate that most chemokines, chemokine receptors, MHC molecules, immunostimulators, and immunoinhibitors were stronger in ICC than ECC. Among these, CXCL2 was poorly expressed in ICC while the expressions of CCL5, CXCL8, and CXCL9 were high, indicating that there were more immune-related molecules in ICC.

### Differences in immune cells between ICC and ECC

**FIGURE 4:**
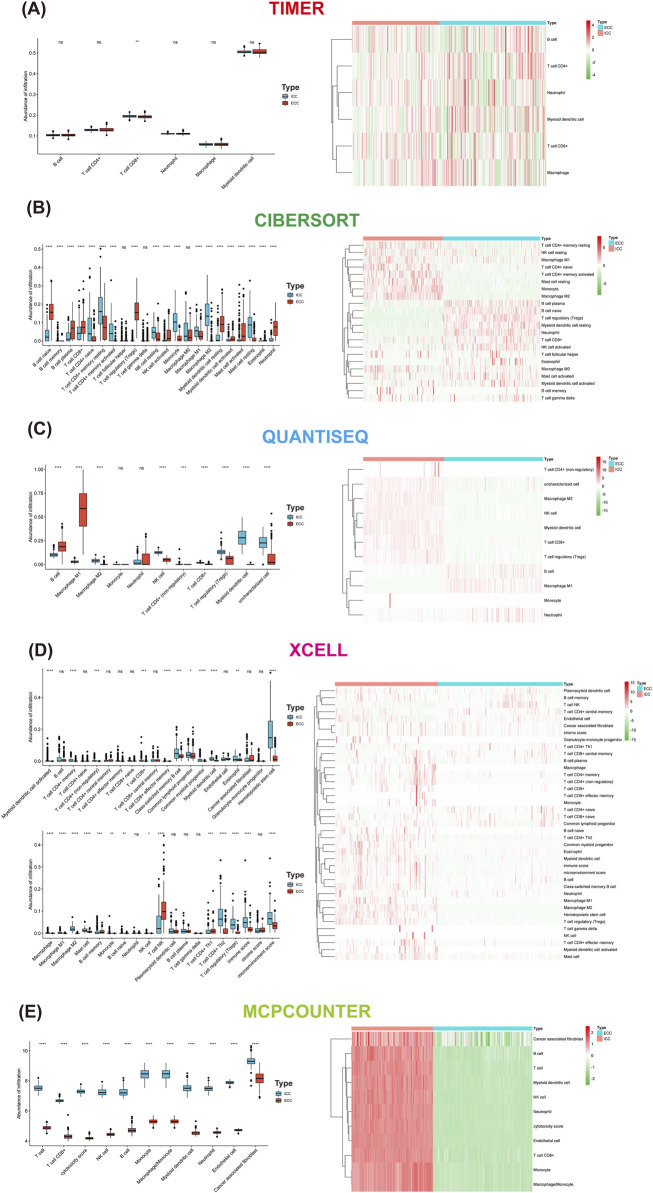
Differences in immune cells between ICC and ECC based on the method used: **(A)** xCell; **(B)** MCP-counter; **(C)** CIBERSORT; **(D)** TIMER; **(E)** quanTIseq. ICC, intrahepatic cholangiocarcinoma; ECC, extrahepatic cholangiocarcinoma.

Five algorithms were used to calculate the differences in immune cells between ICC and ECC. The CD8^+^ T cells were higher in ICC than ECC, except when using CIBERSORT. The M1 macrophages were higher in ICC than ECC based on CIBERSORT and XCELL. The proportion of CD4 memory-activated T cells was higher in ICC than ECC (Figure 4).

### Immune-related differentially expressed genes and pathways between ICC and ECC

**FIGURE 5:**
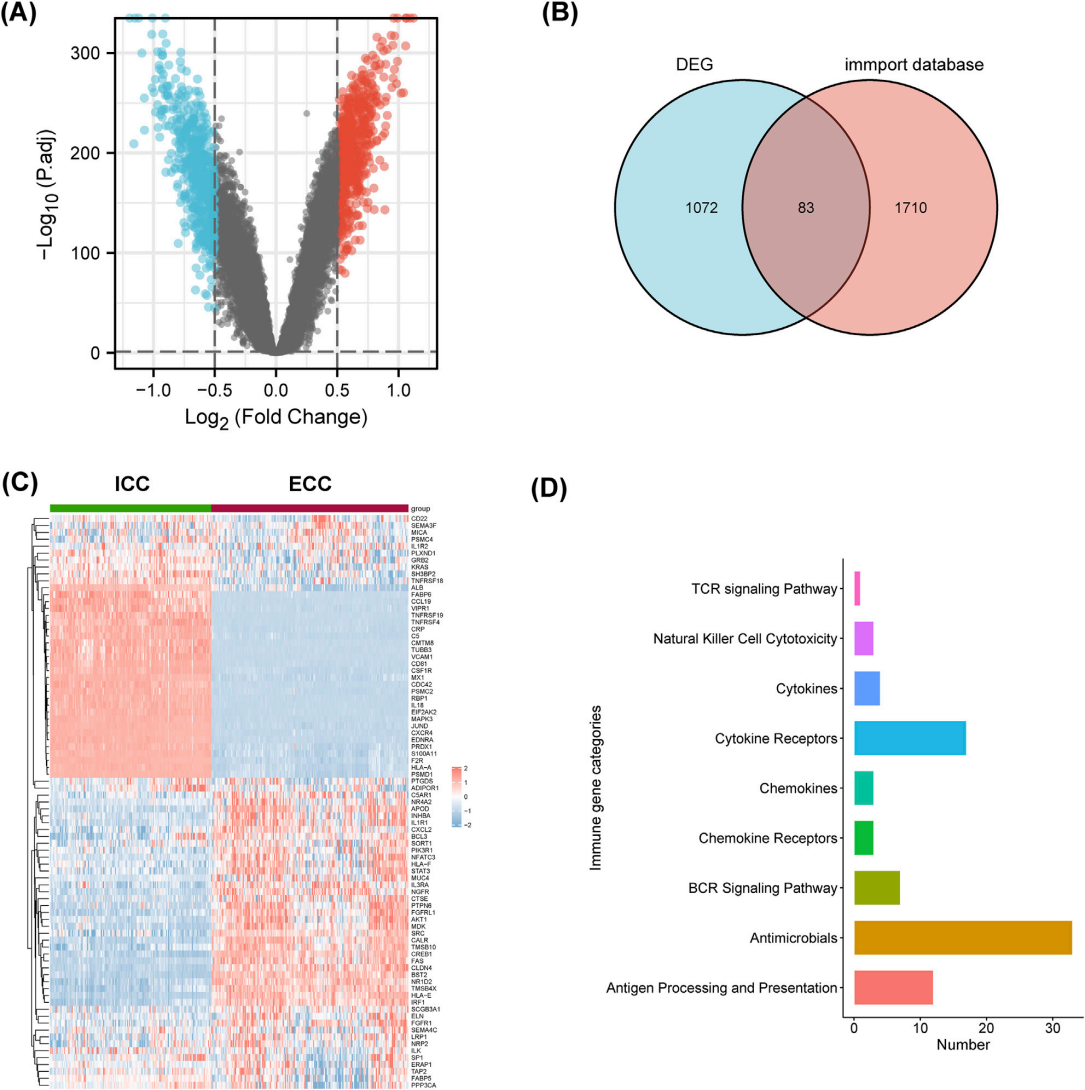
Immune-related differentially expressed genes and pathways were different between ICC and ECC. **(A)** Volcano map of the differentially expressed genes (DEGs); **(B)** intersection of the DEGs and information from the ImmPort database; **(C)** heatmap of the immune-related DEGs between ICC and ECC; **(D)** enrichment analysis of the immune-related DEGs. ICC, intrahepatic cholangiocarcinoma; ECC, extrahepatic cholangiocarcinoma.

Differential analyses of the two normalized datasets using the limma package resulted in 560 upregulated genes in ECC and 590 upregulated genes in ICC (Figure 5A). The 1,155 differential genes of ECC and ICC were intersected with the differential genes from the ImmPort database, and 83 immune-related differentially expressed genes were obtained (Figure 5B).

As shown in Figure 5C, these 83 genes were significantly different between ICC and ECC. The expressions of CCL19, TNFRSF19, TNFRSF4, IL-18, and CXCR4 were higher in ICC patients, while expressions of CXCL2, FAS, and IRF1 were higher in ECC patients. The immune-related differentially expressed genes were mainly enriched in terms of antigen processing and presentation as well as cytokine receptors (Figure 5D). Hence, it is suggested that the differential effects of immunotherapy between ICC and ECC may be related to the immune microenvironment.

## Discussion

This study is a pilot systematic effort to explore the relationships between anatomical classifications of advanced BTCs and ICB responses compared to published results in literature. In our previous study, we found that the PD1-mAb combination therapy was superior to chemotherapy in advanced BTCs (Wang et al., 2022). The present work compares the anti-PD1 effects between ICC and BTCs in other locations. We found that ICC showed better ORR, PFS, and OS than the other BTCs for immunotherapy; however, chemotherapy did not produce significantly different effects between the two groups. Other studies have shown that the efficacy of first- or second-line anti-PD1 therapy for ICC was also significantly better than those for the other BTCs. It was also revealed that patients receiving anti-PD1 treatment showed significantly better OS and PFS than those without anti-PD1 treatment for ICC, while no differences were found for the other BTCs. Therefore, our clinical results show that ICC patients would benefit from immunotherapy.

Previously, the KEYNOTE-966 study examined the efficacy of pembrolizumab combined with a chemotherapy regimen as the first-line treatment for advanced BTCs; subgroup analyses also indicated the potential benefits for patients with ICC (odds ratio (OR) = 0.76, 95% CI: 0.64–0.91) (Kelley et al., 2023). A recent study showed via an unplanned *post hoc* analysis that the benefits of administering PDL1 plus MEK inhibitors were primarily observed in the subgroup of patients with ICC (Yarchoan et al., 2021b). Two other prospective studies with 50 and 32 cases that focused on PD1 combination with chemotherapy or lenvatinib in BTC showed through subgroup analyses that the PFS and ORR did not have statistically significant differences between different primary sites (Li et al., 2022; Lin et al., 2020b), which may be attributed to the small sample size and pending OS. Our study showed that the benefits of anti-PD1 therapy in ICC were greater than those in other BTCs, while there was no significant differences in OS and PFS between the two groups with chemotherapy alone. This means that we could choose immunotherapy based on the primary site in advanced BTCs. Future clinical studies on immunotherapy in BTCs should evaluate the efficacies based on the different primary sites or enroll patients separately by the sites of occurrence. This may be helpful for achieving positive results in BTC with PD1 treatment.

We further investigated the prediction factors of ICB responses between ICC and ECC as well as differences in the immune-related molecules and immune cells between them. Our results showed that the TIDE and dysfunction scores were lower in ICC than ECC. Jiang et al. (2018) showed that a higher TIDE score was associated with not only poor ICB response but also poor survival rates of patients treated with anti-PD1 and anti-CTLA4; meanwhile, high dysfunction scores were also associated with immune resistance. Furthermore, our research showed that CXCL2 was poorly expressed in ICC. Xu et al. (2021) showed that inhibition of CXCL2 or reduction of neutrophils inhibited tumor progression in mouse hepatocellular carcinoma models. Similarly, CXCL2 could promote the proliferation and migration of colon cancer cells in a dose-dependent manner (Lepsenyi et al., 2021). CXCL9 and CXCL10 are important components of T-cell infiltration that activate the chemokine networks and result in a “hot” tumor microenvironment (Reschke and Gajewski, 2022). CCL5 and CXCL9 coexpression also revealed immunoreactive tumors with prolonged survival and responses to ICB (Dangaj et al., 2019). Our immune-related molecule analysis demonstrated upregulation of chemokines associated with CD8^+^ T-cell recruitment in ICC, such as CCL5, CXCL9, and CXCL10, which could be potential causes for the strong immune responses to ICC. Other studies have suggested that CD8^+^ T cells and M1 macrophages are higher in ICC than ECC based on most algorithms. It is well known that CD8^+^ T cells have the ability to selectively eliminate cancer cells and are found in patients with cancers reactive to tumor-specific expressed antigens (Acharya et al., 2020; Philip and Schietinger, 2022). Furthermore, M1 macrophages are thought to promote robust immune responses and clear tumor cells (Choo et al., 2018; Klichinsky et al., 2020). These reported results and the findings of our study suggest that the immune microenvironment may be responsible for the better immune responses observed in ICC.

The genomic features of cholangiocarcinoma are related to its anatomical location and could have therapeutic implications (Lin et al., 2021). We compared the immune-related differential gene expression profiles between ICC and ECC. The expressions of CCL19, IL-18, and CXCR4 were upregulated in ICC patients, while expressions of CXCL2, FAS, and IRF1 were upregulated in ECC patients. IL-18 is upregulated in infiltrating lymphocytes, and blocking it can exert antitumor effects with limited therapeutic efficacy (Becker-Hapak et al., 2021). Zhou et al. (2020) found that decoy-resistant IL-18 exerted antitumor effects by promoting the development of effector T cells, thereby reducing T cell exhaustion and enhancing NK cell activity and maturation. IL-7 and CCL19 are essential for the maintenance of T cell zones in lymphoid organs. In preclinical and clinical studies, CAR-T cells expressing IL-7 and CCL19 were observed to be superior to conventional CAR-T cells in tumor killing (Luo et al., 2020; Pang et al., 2021; Goto et al., 2021). Interestingly, Li et al. (2020) showed that inhibition of CXCR4 alleviated immunosuppression and improved PD1 efficacy. The results of another clinical study suggested that the combination of CXCR4 and PD1 blockade could extend the benefits of chemotherapy in pancreatic cancer (Bockorny et al., 2020). Moreover, pathway enrichment analysis showed that immune-related differentially expressed genes were mainly enriched in terms of antigen processing and presentation as well as cytokine receptors. These results indicate that ICC has a unique immune microenvironment and molecular characteristics, which may be favorable for immunotherapy.

In this study, it was found that anti-PD1 agents had different efficacies for different primary sites of BTCs; however, these findings should be considered from the perspective of some limitations. As a retrospective study, our research is inherently subject to certain biases, particularly those related to loss of patient follow-up, which can lead to follow-up bias. Additionally, the sample sizes for patients with ECC and GBC who received immunotherapy were relatively low. This limited sample size primarily resulted from the lower incidence of ECC and GBC compared to ICC in China. Furthermore, our analysis of the immune microenvironment differences between ICC and ECC relied on data from public databases. These databases, although valuable, are affected by batch effects and other inconsistencies in sample processing and detection. Such issues may introduce variability that could affect our conclusions regarding different immune responses and the potential reasons for varied efficacy of immunotherapy. We recognize that these limitations could also highlight the directions for future prospective studies.

In conclusion, our findings show that the benefits of anti-PD1 agents are greater in ICC than in other advanced BTCs, providing the possibility of immunotherapy as an option based on the anatomical locations of the BTCs. We also reveal that the immune microenvironment and molecular characteristics differ between ICC and ECC and that these could indicate better responses to ICB and longer survival in ICC. Thus, it is feasible to select immunotherapy treatment based on the anatomical location to improve patient outcomes in advanced BTCs.

## Data Availability

The original contributions presented in the study are included in the article/Supplementary Material; further inquiries can be directed to the corresponding authors.
