# Supplementary material for: Anatomical classification of advanced biliary tract cancer predicts programmed cell death protein 1 blockade efficacy
Source: Front Pharmacol. 2024 Aug 30;15:1375769. doi: 10.3389/fphar.2024.1375769 (PMC11392842; doi:10.3389/fphar.2024.1375769)
Supplement: Supplementary file 1 [file DataSheet1.docx]

| **Efficacy Variable** | **ICC (n=90)** | **Others (n=26)** | ***P* value** |
| --- | --- | --- | --- |
| **Overall survival** |  |  |  |
| Median overall survival — months (95% CI) | 15.1(13.2-18.5) | 10.7 (7.0-13.2) | 0.02 |
| Survival rate — % (95% CI) |  |  |  |
| 6 months | 91.1 (85.4-97.2) | 71.9 (56.2-92.0) |  |
| 12 months | 61.1 (52.5-72.8) | 29.6 (15.9-55.0) |  |
| 18 months | 36.7 (28.3-48.9) | 16.9 (6.9-41.3) |  |
| **Progression-free survival** |  |  |  |
| Median progression-free survival — months (95% CI) | 9.5 (8.4-10.6) | 6.2 (3.4-10.2) | 0.02 |
| Rate of progression-free survival — % (95% CI) |  |  |  |
| 6 months | 74.4 (65.8-84.0) | 53.3(37.1-76.7) |  |
| 12 months | 31.2 (22.9-42.6) | 8.2 (2.2-30.9) |  |
| 18 months | 18.0 (11.5-28.3) | 4.1 (0.6-27.9) |  |
| **Response** |  |  |  |
| Objective response rate |  |  | 0.35 |
| No. of patients with a response | 28 | 5 |  |
| % (95% CI) | 31.1 (21.8-41.7) | 19.2 (6.6-39.4) |  |
| Disease control rate |  |  | 0.77 |
| No. of patients with a response | 77 | 21 |  |
| % (95% CI) | 85.6(76.6-92.1) | 80.8(60.6-93.4) |  |
| Best response — no. (%) |  |  | 0.71 |
| Complete response | 2(2.2) | 0 |  |
| Partial response | 26(28.9) | 5 (19.2) |  |
| Stable disease | 49 (54.4) | 16 (61.6) |  |
| Progressive disease | 13 (14.5) | 5 (19.2) |  |

Supplemental Table1 Efficacy Variable of Advanced BTC Treated with anti-PD1

ICC：intrahepatic cholangiocarcinoma

Others (including extrahepatic cholangiocarcinoma and gallbladder cancer)

| **Efficacy Variable** | **ICC (n=70)** | **Others (n=19)** | ***P* value** |
| --- | --- | --- | --- |
| **Overall survival** |  |  |  |
| Median overall survival — months (95% CI) | 16.0(13.5-19.3) | 11.8 ( 8.2-14.5) | 0.04 |
| Survival rate — % (95% CI) |  |  |  |
| 6 months | 91.4 (85.0-98.2) | 78.0 (61.1-99.6) |  |
| 12 months | 65.2 (54.9-77.5) | 33.4 (17.4-64.2) |  |
| 18 months | 39.2 (29.1-52.9) | 22.3 (9.4-52.9) |  |
| **Progression-free survival** |  |  |  |
| Median progression-free survival — months (95% CI) | 7.3 (6.1-8.8) | 5.3 (3.0-10.7) | 0.10 |
| Rate of progression-free survival — % (95% CI) |  |  |  |
| 6 months | 60.0 (49.6-72.6) | 42.1(24.9-71.3) |  |
| 12 months | 15.7 (9.1-27.0) | 10.5 (2.8-39.0) |  |
| 18 months | 5.7 (2.2-14.8) | 5.3 (2.8-39.0) |  |
| **Response** |  |  |  |
| Objective response rate |  |  | 0.26 |
| No. of patients with a response | 21 | 3 |  |
| % (95% CI) | 30.0 (19.6-42.1) | 15.8 (3.4-39.6) |  |
| Disease control rate |  |  | 0.49 |
| No. of patients with a response | 60 | 15 |  |
| % (95% CI) | 85.7(75.3-92.9) | 78.9(54.4-93.9) |  |
| Best response — no. (%) |  |  | 0.59 |
| Complete response | 1(1.4) | 0 |  |
| Partial response | 20(28.6) | 3 (15.8) |  |
| Stable disease | 39 (55.7) | 12 (63.2) |  |
| Progressive disease | 10 (14.3) | 4 (21.0) |  |

Supplemental Table2 Efficacy Variable of Advanced BTC Treated with first line anti-PD1

ICC：intrahepatic cholangiocarcinoma

Others (including extrahepatic cholangiocarcinoma and gallbladder cancer)

Supplemental Table3 Efficacy Variable of Advanced BTC Treated with second line PD1

| **Efficacy Variable** | **ICC (n=20)** | **Others (n=7)** | ***P* value** |
| --- | --- | --- | --- |
| **Overall survival** |  |  |  |
| Median overall survival — months (95% CI) | 11.9 (7.6-NA) | 6.1 (2.4-NA) | 0.02 |
| Survival rate — % (95% CI) |  |  |  |
| 6 months | 85.0 (70.7-100) | 38.1 (13.7-NA) |  |
| 12 months | 45.0 (27.7-73.1) | 19.0 (3.4-NA) |  |
| **Progression-free survival** |  |  |  |
| Median progression-free survival — months (95% CI) | 6.1 (4.1-NA) | 2.6 (1.6-NA) | 0.00 |
| Rate of progression-free survival — % (95% CI) |  |  |  |
| 3 months | 80.0 (64.3-99.6) | 38.0(13.7-NA) |  |
| 6 months | 50.0 (31.2-77.3) | NA |  |
| **Response** |  |  |  |
| Objective response rate |  |  | 1.00 |
| No. of patients with a response | 6 | 2 |  |
| % (95% CI) | 30.0 (11.9-54.3) | 28.5 (3.7-71.0) |  |
| Disease control rate |  |  | 1.00 |
| No. of patients with a response | 17 | 6 |  |
| % (95% CI) | 85.0(63.1-96.8) | 85.7(42.1-99.6) |  |
| Best response — no. (%) |  |  | 1.00 |
| Complete response | 1(5.0) | 0 |  |
| Partial response | 5(20.0) | 2 (28.5) |  |
| Stable disease | 11 (55.0) | 4 (57.2) |  |
| Progressive disease | 3 (15.0) | 1 (14.3) |  |

ICC：intrahepatic cholangiocarcinoma

Others (including extrahepatic cholangiocarcinoma and gallbladder cancer)

Supplemental Table 4 Clinical Characteristics for different sites of advanced BTC Treated with anti-PD1

|  | **ICC (n=90)** | **ECC (n=10)** | **GBC (n=16)** | ***P* value** |  |
| --- | --- | --- | --- | --- | --- |
| **Gender(%)** |  |  |  | 0.061 |  |
| Female | 34 (37.8) | 1 (10.0) | 9 (56.2) |  |  |
| Male | 56(62.2) | 9 (90.0) | 7 (43.8) |  |  |
| **Age**—**years old** | 55.00(47.25-62.75) | 59.50 (51.75, 67.50) | 59.50(54.75, 66.00) | 0.163 |  |
| **HBV infection**—**(n , %)** |  |  |  | 0.188 |  |
| Positive | 32(36.4) | 3 (30.0) | 2 (12.5) |  |  |
| Negative | 56(63.6) | 7 (70.0) | 14 (87.5) |  |  |
| **Pathological Grade**  **—(n , %)** |  |  |  | 0.546 |  |
| Low | 16(25.0) | 0 (0.0) | 2 (12.5) |  |  |
| Low to moderately | 18(28.1) | 3 (30.0) | 5 (31.2) |  |  |
| Moderately | 29(45.3) | 3 (30.0) | 2 (12.5) |  |  |
| Well | 1(1.6) | 4 (40.0) | 6 (37.5) |  |  |
| Unknown | 38(26.8) | 0 (0.0) | 1 (6.2) |  |  |
| **Clinical Stage—(n , %)** |  |  |  | 0.723 |  |
| IIIB or IIIC | 37(41.1) | 5(50.0) | 8(50.0) |  |  |
| IV | 53(58.9) | 5(50.0) | 8(50.0) |  |  |
| **Metastatic—(n , %)** |  |  |  | 0.661 |  |
| Yes | 47(52.2) | 4(40.0) | 7(43.8) |  |  |
| No | | 43(47.8) | 6(60.0) | 9(56.2) |  |

ICC：intrahepatic cholangiocarcinoma

ECC：extrahepatic cholangiocarcinoma

GBC：gallbladder cancer

Supplementary Figure1


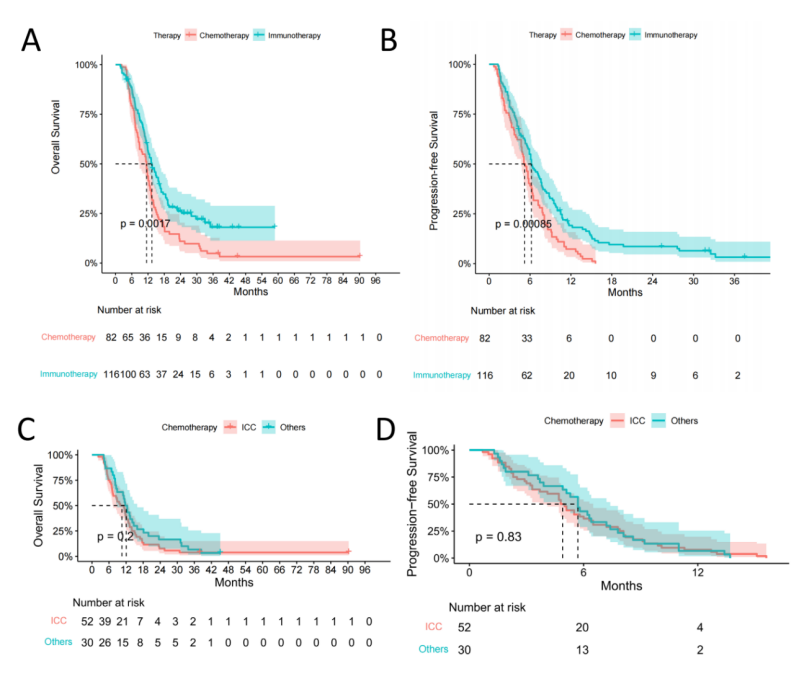


Supplementary Figure 1. Survival analysis. (A-B) Kaplan-Meier estimates of OS and PFS for chemotherapy alone and combination therapy containing anti-PD1 in advanced BTC. (C-D) Kaplan-Meier estimates of OS and PFS for chemotherapy in ICC and Others. OS: overall survival; PFS: progression-free survival; BTC: biliary tract cancer; ICC: intrahepatic cholangiocarcinoma; Others: extrahepatic cholangiocarcinoma and gallbladder cancer.

Supplementary Figure 2


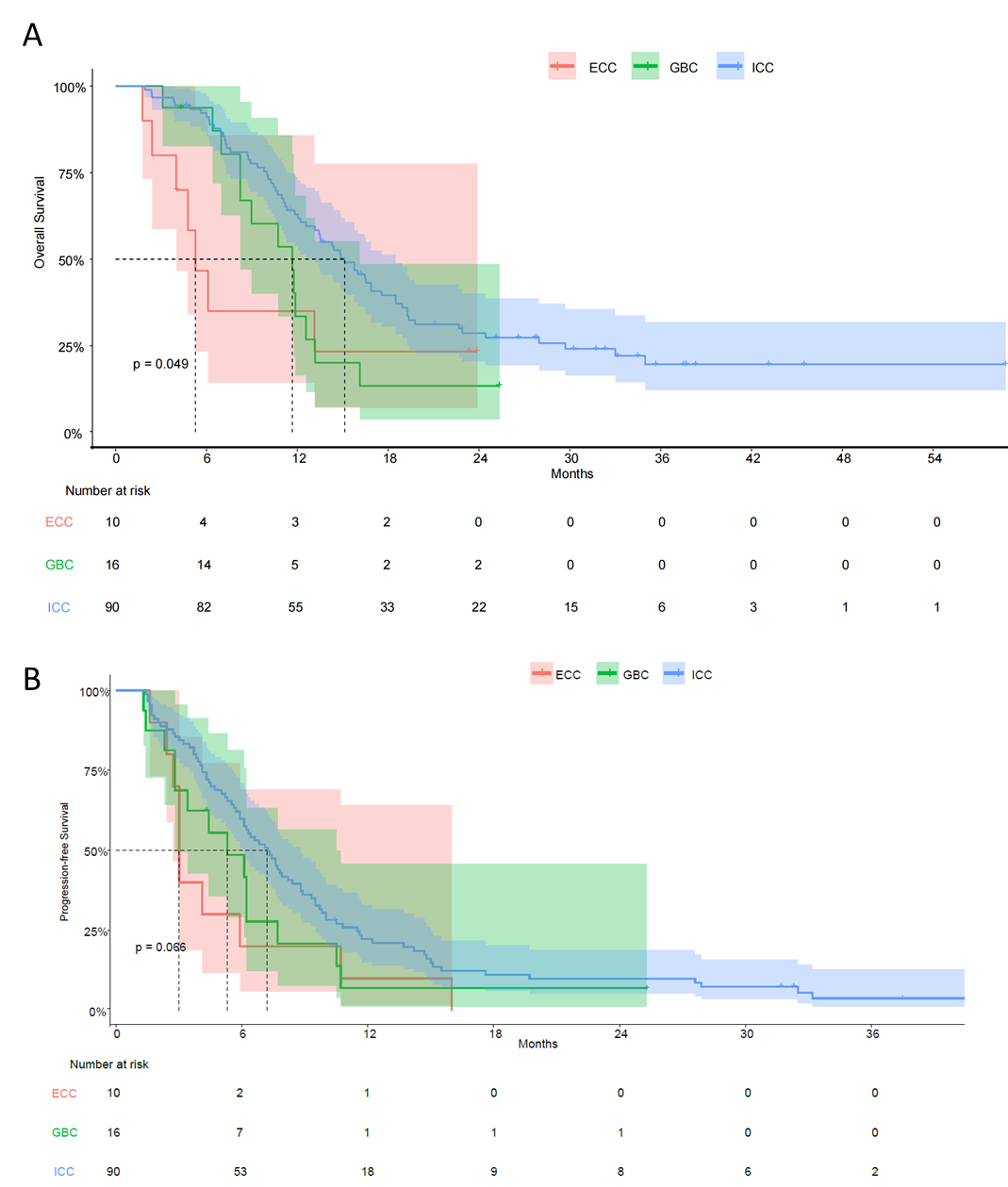


Supplementary Figure 2. Survival analysis stratified by immunotherapy. Kaplan-Meier estimates of OS and PFS for anti-PD1 in ICC、ECC and GBC.
